# Functioning Mediastinal Paraganglioma Associated with a Germline Mutation of von Hippel-Lindau Gene

**DOI:** 10.3390/jcm7060116

**Published:** 2018-05-23

**Authors:** Thibault Bahougne, Pauline Romanet, Amira Mohamed, Kevin Caselles, Thomas Cuny, Anne Barlier, Patricia Niccoli

**Affiliations:** 1 Service d’Endocrinologie et Diabète, Hôpitaux Universitaires de Strasbourg, 67000 Strasbourg, France; 2 Institut des Neurosciences Cellulaires et Intégratives, CNRS (UPR 3212), 67000 Strasbourg, France; 3 Laboratory of Molecular Biology, Hospital la Conception, Aix-Marseille Université, INSERM, MMG, 13005 Marseille, France; pauline.romanet@ap-hm.fr (P.R.); amira.mohamed@ap-hm.fr (A.M.); anne.barlier@univ-amu.fr (A.B.); 4 Laboratoire d’Anatomie et Cytologie Pathologique, Hôpital Nord, Chemin des Bourrely, 13915 Marseille, CEDEX 20, France; kevin.caselles@ap-hm.fr; 5 Department of Endocrinology, Conception Hospital, Centre de Référence des Maladies Rares Hypophysaires HYPO, INSERM U1251, Marseille Medical Genetics, Aix-Marseille Université and AP-HM, 13005 Marseille, France; thomas.cuny@ap-hm.fr; 6 Oncologie Medical Department, IPC, 13009 Marseille, France; niccolip@ipc.unicancer.fr

**Keywords:** hemangioblastoma, hypertension, paraganglioma, von Hippel-Lindau disease

## Abstract

We report the case of a 21-year old woman presenting with high blood pressure and raised normetanephrine levels. Indium-111-pentetreotide single photon-emission computed tomography with computed tomography (SPECT/CT) and 2-deoxy-2-[fluorine-18]fluoro-d-glucose (FDG) positron emission tomography/computed tomography (PET/CT) imaging showing isolated tracer-uptake by a 2 cm tumor close to the costovertebral angle of the third thoracic vertebra. Thoracic surgery led to normalization of normetanephrine levels. Histological findings were consistent with the presence of a paraganglioma. Mutations in *SDHA*, *SDHB*, *SDHC*, *SDHD*, *RET*, *SDHAF2*, *TMEM127*, *MAX*, *NF1*, *FH*, *MDH2*, and *EPAS1* were absent, but a heterozygous missense mutation, c.311G > T, was found in exon 1 of the von Hippel-Lindau gene, *VHL*, resulting in a glycine to valine substitution in the VHL protein at position 104, p.Gly104Val. This same mutation was found in both the mother and the 17-year old sister in whom a small retinal hemangioblastoma was also found. We diagnose an unusual functional mediastinal paraganglioma in this young patient with a germline *VHL* gene mutation, a mutation previously described as inducing polycythemia and/or pheochromocytoma but not paraganglioma or retinal hemangioblastoma.

## 1. Introduction

Phaeochromocytomas (PHEO) and extra-adrenal paragangliomas (PGL) are functional neuroendocrine tumors associated with a clinically significant increase of catecholamine plasma levels. Functioning PGL usually develop in the abdomen [1]. Today, germline mutations in a panel of 15 susceptibility genes are found in up to a third of PHEO/PGL cases, as such these tumors display one of the strongest hereditary backgrounds [2]. Overall, a PHEO or PGL will occur in a range of 50% of the individuals carrying a germline mutation in any of these genes [3].

## 2. Materials and Methods

The coding exons and flanking regions of genes implicated in PHEO/PGL (MYC associated factor X (*MAX*; NM_002382.3), rearranged during transfection tyrosine kinase receptor gene (*RET*; NM_020975.4), succinate dehydrogenase subunit A (*SDHA*; NM_004168.3), succinate dehydrogenase complex assembly factor 2 (*SDHAF2*; NM_017841.2), succinate dehydrogenase subunit B (*SDHB*; NM_003000.2), succinate dehydrogenase subunit C (*SDHC*; NM_003001.3), succinate dehydrogenase subunit D (*SDHD*; NM_003002.2), transmembrane protein 127 (*TMEM127*; NM_017849.3), fumarate hydratase (*FH*; NM_000143.3), neurofibromatosis type I (*NF1*; NM_001042492.2), malate dehydrogenase 2 (*MDH2*; NM_005918.3), Endothelial PAS domain protein 1 (*EPAS1*; exon 9 and 12, NM_001430) and von Hippel-Lindau gene (*VHL*; NM_000551.3)) were analyzed using a combination of next-generation sequencing (NGS, Illumina Miseq TSCA Librairies) and multiplex ligand probe assay (MLPA). The allelic variants found by NGS were confirmed using Sanger method. In silico analysis of variants was performed using Polyphen2 (http://genetics.bwh.harvard.edu/pph2/), UMD-predictor^®^ [4], and ALAMUT 2.2.0 (http://www.interactive-biosoftware.com/) software. Blood samples for genomic DNA analysis were taken after written informed consent and patient gave written (signed) informed consent after being given a detailed explanation of the publication. Genetic researches were carried out according to The Code of Ethics of the World Medical Association (Declaration of Helsinki).

## 3. Result

**Figure 1:**
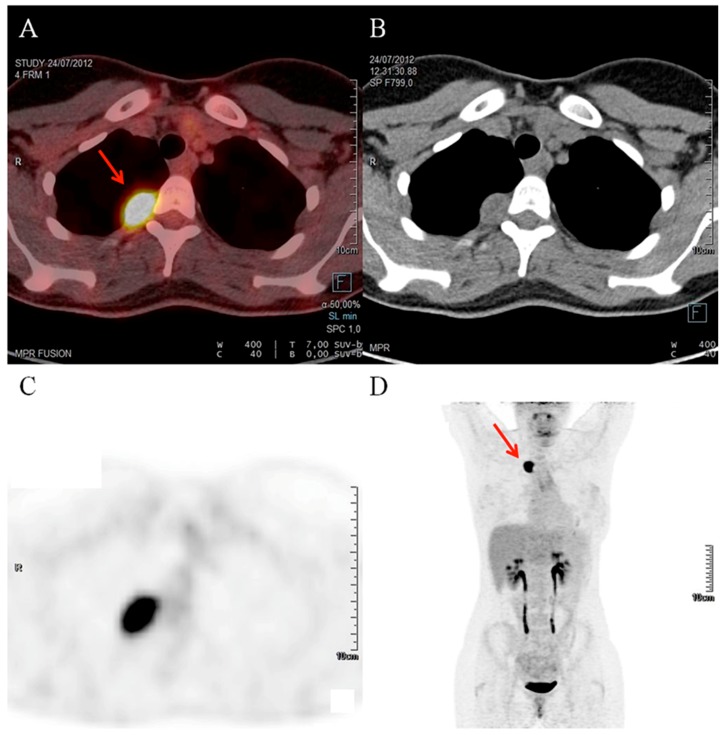
2-deoxy-2-[fluorine-18]fluoro-d-glucose (FDG) positron emission tomography/computed tomography (PET/CT). (**A**,**C**,**D**) Isolated hypermetabolism (standard uptake value up to 18 SUV max) of a 2 cm tumor (arrows), close to the costovertebral angle of the third right thoracic vertebra. Note the absence of other uptake. (**A**) coronal fused PET/CT images; (**B**) planar view showed a 2 cm para-vertebral lesion; (**C**) anterior whole-body maximum intensity projection (MIP) images.

A healthy 21 year-old woman without past medical history was referred to the department of endocrinology for high blood pressure, suspicious of endocrine hypertension. She described palpitation episodes over the previous six months. After primary hyperaldosteronism and Cushing syndrome exclusion, a four-fold increase in normetanephrine levels was observed in both the plasma and urinary samples (Table 1) but adrenal glands on computed tomography (CT) scan were normal. Finally, an isolated uptake of the radiotracer by a 2 cm lesion close to the costovertebral angle of the third right thoracic vertebra was detected by Indium-111-pentetreotide SPECT/CT and 2-deoxy-2-[fluorine-18]fluoro-d-glucose (FDG) positron emission tomography/computed tomography (PET/CT), respectively (Figure 1). After calcium channel blocker pretreatment, the patient underwent a surgical resection of the lesion, which resulted in normalization of blood pressure and normetanephrine levels in the postoperative period.

**Figure 2:**
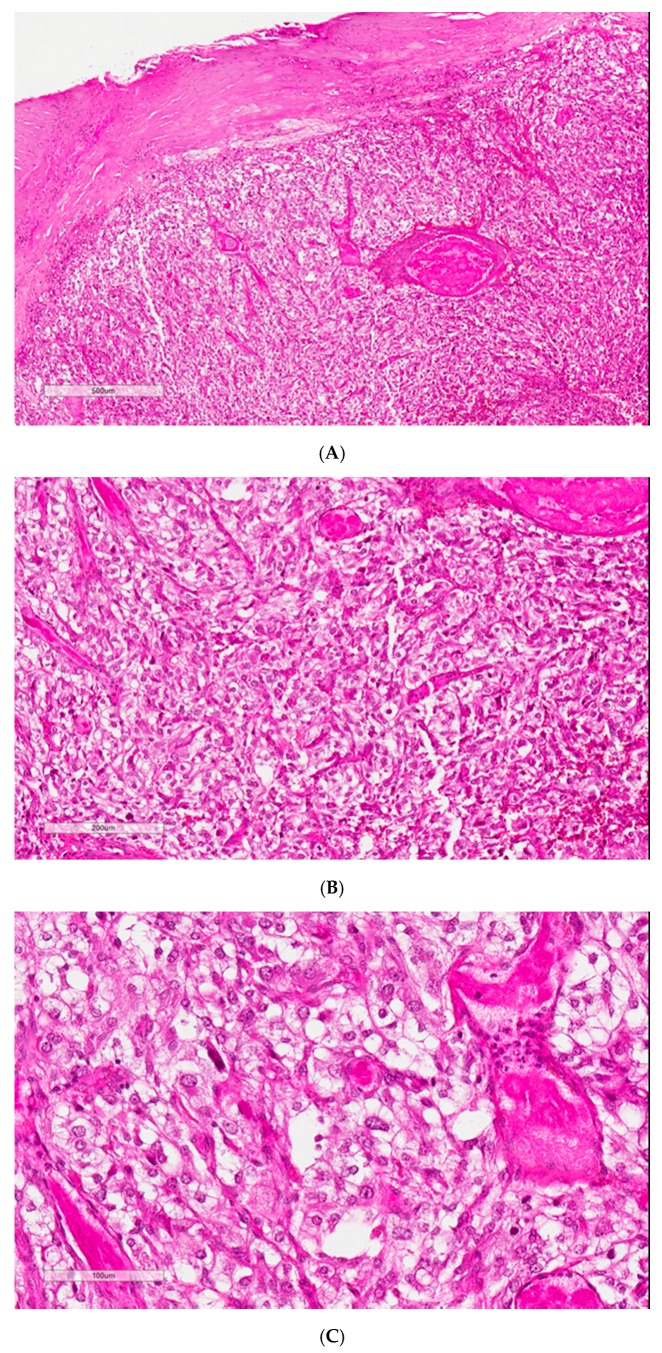
Three microns paraffin-embedded PGL section stained with HPS (hemalun, phloxin, saffron). (**A**) low magnification with PGL capsule (X2); (**B**) PGL architecture in nests and cords (X10); (**C**) PGL cells with finely nucleated rounded nuclei developed in a richly vascularized stroma. PGL is well limited by a fibrous capsule. Homogeneous proliferation with nests, lobules, and cords architecture made by large cells with clear or eosinophilic cytoplasm.

Histological findings showed a 2 × 1.8 × 1.5 cm (2.8 cm^3^) lesion consistent with a paraganglioma. This tumor showed homogeneous proliferation with trabecular, acinar, and alveolar architecture. It consisted of large cells with clear or eosinophilic cytoplasm, characterized by a richly vascularized, nested organization. Depending on the localization, the nuclei could be central or irregular. The stroma was highly vascularized and congestive, and some hemorrhagic localization of variable density connective tissue was found on the periphery. The PASS score and the Ki67 data were not available (Figure 2).

Blood samples for genomic DNA analysis were taken after written informed consent had been received. While screening for mutations in *SDHA*, *SDHB, SDHC*, *SDHD*, *RET*, *SDHAF2*, *TMEM127*, *MAX*, *NF1*, *FH*, *MDH2*, and *EPAS1* genes was negative, a heterozygous missense variation in exon 1 of the von Hippel-Lindau (*VHL)* disease gene was found. This c.311G > T mutation gives rise to an amino acid substitution of valine by glycine at position 104 in the VHL protein, p.Gly104Val. Large VHL deletions were also excluded by MLPA. Our variant is localized on a normally fairly well conserved nucleotide and amino acid. This variant was not found on both the ExAC browser (http://exac.broadinstitute.org/) and Exome Variant Server (http://evs.gs.washington.edu/EVS/). In silico analysis classified this variant as probably damaging.

The staging, including ophthalmologic exam, thoraco-abdomino-pelvic CT, cerebral and medullar MRI, was normal. The heterozygous missense variant for *VHL* gene was identified on her 17-year-old sister and her mother, but not her father. The sister´s evaluation was normal with the exception of a small retinal hemangioblastoma. The patient’s mother had refused a complete staging. Polycythemia was not observed in the explored patients.

## 4. Discussion

Von Hippel-Lindau (VHL) disease is an autosomal dominant disorder with an estimated prevalence in the population of 1/53,000 individuals, whose diagnosis requires one of the following criteria: (i) more than one hemangioblastoma in the central nervous system (CNS) or retina; (ii) a single hemangioblastoma in the CNS or retina plus a visceral complication; (iii) any one of the above manifestations combined with a family history; or (iv) systematic family screening after discovery of a *VHL* propositus [5]. Two types of VHL disease are described based on the risk of developing PHEO or PGL: type 1—low risk, and type 2—high risk. Type 2 can be further subdivided, based on the additional risk of developing renal cell carcinoma (RCC), into: type 2A—low risk, type 2B—high risk, and type 2C—no additional risk than from PHEO or PGL [6]. The earliest and most common manifestations of VHL disease, being present in 80% of cases, are retinal or CNS hemangioblastomas (of types 2A and 2B). On the contrary, PGL are quite uncommon in VHL disease, tumors mainly occur in the adrenals and bilateral PHEO are found in 50% of the case. A gradient of predisposition depending on VHL loss of function degree has been suggested in RCC risk. During lifetime, RCC can occurred in 3 (type 2A) to 75% (type 2B and 1) of VHL patients and are the primary cause of death [2]. The follow-up for this family has been too short to conclude anything except a diagnosis of VHL disease of type 2A or 2B.

In this case, the noradrenergic PGL profile clearly indicated a higher likelihood of VHL or SDH mutations [2]. VHL and SDH were first analyzed, then a larger gene panel was analyzed in order to exclude other hypotheses given their atypical VHL phenotype. Practitioners should be aware that NGS multiple gene analysis without clinical orientation exposes them to discovering non-pathogenic or variants of unknown significance.

The *VHL* gene is a tumor-suppressor gene located on the short arm of chromosome 3 (3p25.3). The expressed protein, pVHL, modulates the ubiquitination and subsequent destruction of HIF1alpha, hypoxia-inducible factor 1, subunit alpha, the main regulator of gene-expression in hypoxic cells [7]. In this family, the missense mutation on exon 1 of *VHL* resulted in a p.Gly104Val substitution which has not been described so far in the literature as being causative of PGL or hemangioblastoma. Nevertheless, for the following reasons we came to the diagnostic conclusion that this family had VHL disease namely: (i) we found a rare *VHL* gene mutation resulting in the substitution of a valine for a glycine at amino acid 104 in the VHL protein, a moderately well conserved amino acid; (ii) a review of cases of VHL disease showed that other missense mutations for the same amino acid have been reported [8]; (iii) the same *VHL* gene variant has been described in a patient with PHEO, probably of VHL-type2C, where there was also an attenuated familial disease phenotype possibly due to a limited loss-of-function of pVHL [9]; (iv) this variant has been reported in a patient with congenital polycythemia [10] and eventually, the same gene variant was identified in our patient’s younger, 17 year-old, sister who was found to have a retinal hemangioblastoma.

The heterogeneous phenotype observed with this heterozygous missense mutation (c.311G > T) in the two sisters could be due to the additive effect of a second, unknown, pathogenic germline mutation elsewhere on the genome inducing a severe pVHL loss-of-function [6,9]. A phenotype such as the one in our family could possibly result from a haplotype transmission of a mutation elsewhere impairing the interaction of pVHL on *HIF1alpha* leading to a reduced degradation of pHIF1 [7]. In this latter study, different *VHL* mutations led to gradual loss-of-function of the gene which might explain the existence of different phenotypes [6].

Follow-up of all the family members has continued for almost five years with no sign of relapse in the patient. Follow-up must be continued for her lifetime because of the potential risk of developing a RCC or PHEO from this *VHL* mutation [11].

For this family, according to phenotype, diagnosis hypothesis (VHL or SDH) was right. If syndromic presentation is suspected, targeted genes could be analyzed by Sanger method. If no pathogenic mutation is found, NGS targeting gene panel should be realized. In non-syndromic situations, NGS targeting gene panel should be firstly proposed. After identifying a variant of unknown significance, the data from genome-metabolome-phenome provide tools to classify of variants as pathogenic or benign [2].

In conclusion, we report the first diagnosis of a functional mediastinal paraganglioma in a young patient with a missense mutation in exon 1 of the *VHL* gene. Until now, this mutation has been described in the literature with cases of polycythemia and/or PHEO but not paraganglioma or retinal hemangioblastoma. The association of PGL with retinal hemangioblastoma in these two sisters with the same c.311G > T mutation prompted us to diagnose familial VHL syndrome. 

## Figures and Tables

**Table 1:** Biological data of the 21-year-old woman with high blood pressure.

Hormones	Results		Normal Laboratory Ranges
	Presurgical	2 Months Post-Surgical	
Plasma aldosterone			
Lying down	11		10 to 105 pg/mL
Standing	323		34 to 273 pg/mL
Plasma renin			
Lying down	5		<20 pg/mL
Standing	17.40		5 to 40 pg/mL
Chromogranin A	128	74	27 to 94 ng/mL
Plasma			
Normetanephrine	3.74	0.68	<0.94 nmol/L
Metanephrine	0.12	0.14	<0.37 nmol/L
24-h urine			
Normetanephrine	1795		53 to 391 µg/24 h
Metanephrine	90		39 to 284 µg/24 h
